# Sequence-based detection of emerging antigenically novel influenza A viruses

**DOI:** 10.1098/rspb.2024.0790

**Published:** 2024-08-14

**Authors:** Alpha Forna, K. Bodie Weedop, Lambodhar Damodaran, Norman Hassell, Rebecca Kondor, Justin Bahl, John M. Drake, Pejman Rohani

**Affiliations:** ^1^ Odum School of Ecology, University of Georgia, Athens, GA 30602, USA; ^2^ Center for the Ecology of Infectious Diseases, University of Georgia, Athens, GA 30602, USA; ^3^ Department of Epidemiology and Biostatistics, College of Public Health, University of Georgia, Athens, GA 30606, USA; ^4^ Centers for Disease Control and Prevention, Atlanta, GA 30329, USA; ^5^ Center for Influenza Disease & Emergence Research (CIDER), Athens, GA 30602, USA; ^6^ Department of Infectious Diseases, University of Georgia, Athens, GA 30602, USA

**Keywords:** antigenic transition, infectious disease forecasting, influenza virus, unsupervised machine learning, viral evolution

## Abstract

The detection of evolutionary transitions in influenza A (H3N2) viruses’ antigenicity is a major obstacle to effective vaccine design and development. In this study, we describe Novel Influenza Virus A Detector (NIAViD), an unsupervised machine learning tool, adept at identifying these transitions, using the HA1 sequence and associated physico-chemical properties. NIAViD performed with 88.9% (95% CI, 56.5–98.0%) and 72.7% (95% CI, 43.4–90.3%) sensitivity in training and validation, respectively, outperforming the uncalibrated null model—33.3% (95% CI, 12.1–64.6%) and does not require potentially biased, time-consuming and costly laboratory assays. The pivotal role of the Boman’s index, indicative of the virus’s cell surface binding potential, is underscored, enhancing the precision of detecting antigenic transitions. NIAViD’s efficacy is not only in identifying influenza isolates that belong to novel antigenic clusters, but also in pinpointing potential sites driving significant antigenic changes, without the reliance on explicit modelling of haemagglutinin inhibition titres. We believe this approach holds promise to augment existing surveillance networks, offering timely insights for the development of updated, effective influenza vaccines. Consequently, NIAViD, in conjunction with other resources, could be used to support surveillance efforts and inform the development of updated influenza vaccines.

## 1. Introduction

With more than 3 million cases of severe illness per year and around half a million annual deaths worldwide, infections with influenza A viruses (e.g. H3N2, H1N1) are a leading cause of morbidity and mortality in humans, as well as a significant economic burden [1]. As part of holistic public health policies aimed at reducing the burden of influenza, experimental and computational approaches have determined that viral antigenic drift [2] leads to sporadic dominance of new antigenic clusters and subsequently the need for updated vaccines [3–5]. The critical challenge is to establish whether there is an antigenic mismatch between cocirculating viruses and those responsible for existing immunity, including both vaccine derived and infection derived. Inhibition-based laboratory assays are regarded as the current standard for identifying drifted viruses, but these assays are retrospective, could be biased, time-consuming and the haemagglutination-inhibition (HI) assay in particular can be too labour-intensive for routine utilization at large scale unlike high content imaging-based neutralization (HINT) assays [4,6]. More recently, computational methods have been used to identify antigenic variants of influenza A [4,7–9]. For instance, it has been shown that surface glycoprotein HA1 amino acid sequences may be mapped to HI titre values and provide a measure of viral drift [6,10,11]. But existing computational tools require large quantities of serological data to generate accurate predictions about antigenicity [12]. With the ubiquitous availability of high-throughput nucleotide sequencing technology, sampling of viral surface protein amino acid sequences now far outpaces corresponding measurements of HI titres. Thus, if a method could be devised to predict antigenicity without the need for direct measurements of HI titres, gains in predictive accuracy and timely detection and response could be substantial.

Moreover, in recent years, the inability of some influenza A (H3N2) viruses to agglutinate red blood cells has made experimental HI titre used in supervised antigenicity prediction less reliable for prediction [11]. Thus, for H3N2 viruses, there has been a switch to micro-neutralization assays [13] (e.g. focus reduction assay (FRA) and HINT) [11,14] that reduce antigenic mischaracterization resulting from viral adaptation to cell cultures (particularly in instances where cells are inappropriate and protocols for virus isolation and propagation are not followed), but these assays are not yet widely used outside of specialized laboratory settings [11]. Furthermore, although advancements in sequencing technology have improved our ability to identify different viral strains during routine surveillance, it should not be assumed that there will always be sufficient mutant strains from large samples for effective supervised antigenicity prediction. Thus, despite abundant genetic data about cocirculating viruses, one might not be able to perform antigenic assays on these strains due to their low presence. As genomic surveillance continues to expand, this challenge will become increasingly prominent. If sufficiently accurate, unsupervised learning approaches would not only be independent of inhibition assay measurements but would also be more robust to smaller datasets and imbalances in the frequency of antigenic cluster-to-non-cluster transitions, particularly in instances where antigenicity is modelled as a binary outcome of few antigenic transitions compared with more non-antigenic transitions. We, therefore, propose that since evolutionary innovations that result in a cluster transition are comparatively rare, even with extensive differences within antigenic clusters [15,16], one should view the identification of novel variants as a kind of anomaly detection within an unsupervised learning framework.

Isolation forests [17] and one-class support vector machines (SVMs) [18] are examples of unsupervised learning methods that have been used extensively in other domains to detect anomalies without mapping to a targeted outcome during model development [19,20]. In the biological sciences, these techniques have been used extensively for the structural and functional characterization of proteins. For instance, a previous study suggests that scaling unsupervised learning to 250 million protein sequences could reveal biological structure and function using only sequence data [21]. However, such powerful techniques have not yet been applied to virus variant detection, even for benchmark datasets. A review of computational tools used to predict influenza phenotype identified only one study that characterized antigenicity using unlabelled data [22]. Further, noting that protein fold recognition and structural class predictions were made using modelled physico-chemical properties calculated from amino acid sequences [23], we hypothesize that an unsupervised algorithm that makes use of HA1 amino acid sequences and associated physico-chemical properties could enable rapid detection of new antigenic variants of the H3N2 virus.

For this reason, we considered five physico-chemical properties—the Boman’s index, isoelectric point, hydrophobicity, electrostatic charge and instability index—of mechanistic relevance to viral antigenicity [24]. The Boman’s index estimates the potential of peptides/proteins to bind to other proteins [25]. The isoelectric point is the pH at which a protein carries no net electrical charge, relevant to antigenicity as isoelectricity affects the interaction of proteins with other molecules [26]. Hydrophobicity, the tendency of protein regions to repel water, influences protein folding and stability, crucial for the formation of antigenic determinants by exposing or masking potential epitopes [27]. Electrostatic charge affects the formation of immune complexes, with charge interactions influencing the antigenicity [28]. Lastly, the instability index predicts the *in vivo* half-life and thermostability of proteins, impacting their degradation and the presentation of antigenic peptides, key for initiating immune responses [29].

**Figure 1.**
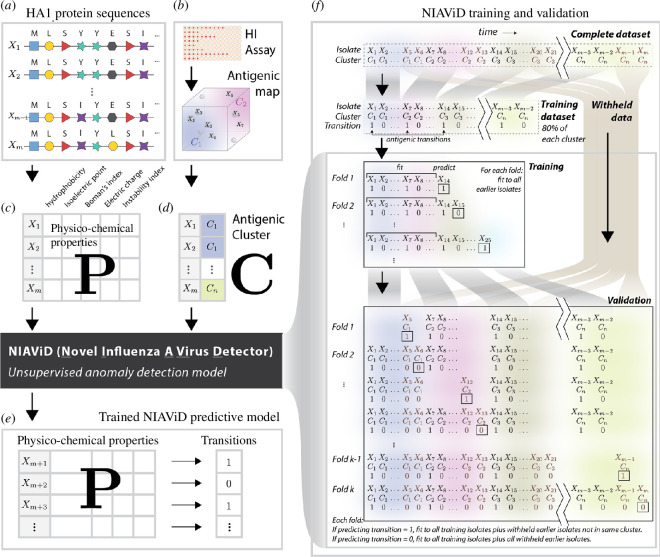
NIAViD is a model for detecting antigenic transitions in influenza A (H3N2) without knowledge of cluster identity. This figure illustrates the computational workflow used to fit and validate NIAViD. Input data for NIAViD are the amino acids at 329 positions from the HA1 region of the haemagglutinin protein for m isolates in the data (*a*). For each isolate, we calculate the average over the 329 amino acid sites for each of five physico-chemical properties—hydrophobicity, isoelectric point, Boman’s index, electric charge and a measure of instability—which constitute the *m* × 5 prediction matrix P (*c*). Prior to model training, isolates were randomly assigned to training and validation datasets after stratifying by cluster. The training was performed iteratively by sequentially introducing each isolate into the analysis (*f*). With each iteration, outlier detection was used to assign an anomaly score for the new isolate with respect to all prior isolates. Isolates that receive a high anomaly score at their first appearance are considered candidates for antigenic transition, i.e. the appearance of a new antigenic cluster. We perform the validation by constructing a sample from all datasets with the validation isolate withheld. Thus, for validation (but not for training), we require information on the antigenic cluster C (*d*) to which each isolate belongs, obtained from haemagglutinin inhibition assays (*b*). For each isolate in the validation data, the cluster identity is examined and compared with all prior isolates in the validation data. If the cluster is new, then that isolate is labelled a ‘validation transition’. Otherwise, it is not a transition. For non-transitions, the input data are all the training data together with any validation data from prior iterations of the validation. For validation transitions, the input data are all the training data less any isolates from the cluster to be predicted (because the model cannot have seen any information on a new cluster) together with any validation data from prior iterations of the validation (*f*). The mapping produces a binary output (*e*). This output can be compared with the true transitions and non-transitions in a confusion matrix. See electronic supplementary material for the pseudocode.

Here, we report on an unsupervised influenza prediction tool—Novel Influenza Virus A Detector (NIAViD) (figure 1)—that takes these physico-chemical covariates derived from HA1 amino acid sequences as input and returns a binary antigenic transition/antigenic non-transition label for each viral sequence. With NIAViD, we model the evolving set of antigenic clusters to demonstrate the sequence-based detection of emerging antigenically novel influenza A (H3N2) viruses. By leveraging existing sequenced-based data streams, our tool can efficiently complement routine virological surveillance for vaccine strain selection. To familiarize readers with the technical terminology used throughout this article, we have provided a glossary of key specialists’ terms in electronic supplementary material, table S1.

## 2. Results

**Figure 2.**
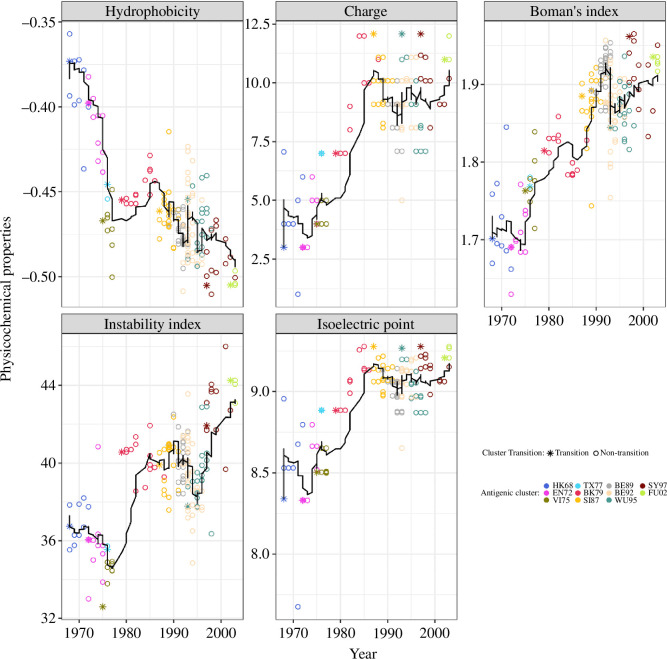
The five physico-chemical covariates of influenza virus A (H3N2) virus HA1 sequences are plotted against the year of isolation, with the 11 antigenic clusters marked by distinct colours and the two cluster transitions marked by different shapes. The horizontal black line in each panel represents the rolling average of each physico-chemical property, calculated using a rolling window of 10 influenza A viruses.

NIAViD is a process (figure 1) to predict the antigenic transition of influenza isolates from five physico-chemical covariates calculated directly from HA1 sequence data (the Boman’s index, isoelectric point, hydrophobicity, electrostatic charge and an instability index) and does not depend on other assay scores (e.g. HAI, FRA and HINT) [11,14,30]. To demonstrate the NIAViD process, we used the 273 H3N2 isolates reported in the classic study of Smith *et al*. [31], which have become a benchmark to compare influenza antigenicity prediction algorithms [6,8,32]. The trends from 1968 to 2003 in each virus physico-chemical covariate are shown in figure 2 and are statistically different from a randomized sample of the same sequences (i.e. all non-parametric Wilcoxon rank-sum test *p*-values comparing the physico-chemical trends are <0.05) (electronic supplementary material, appendix S1, table S2 and figure S1).

**Figure 3.**
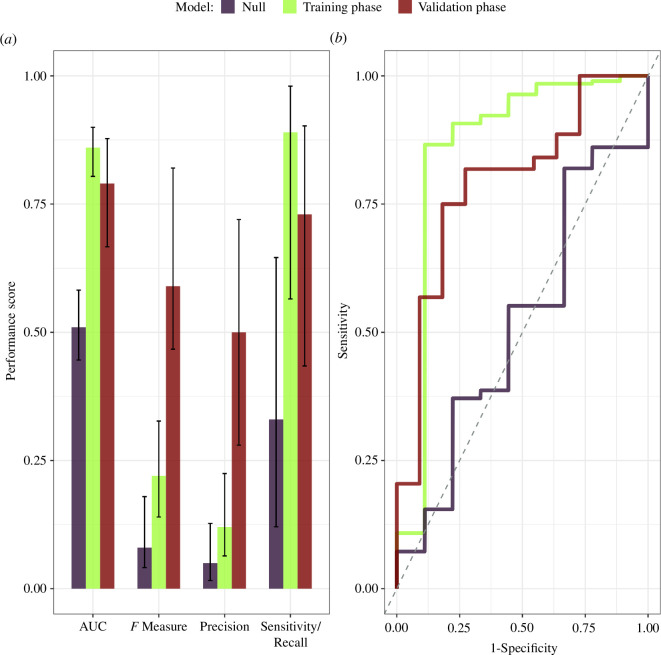
The null, training and validation performance of NIAViD. Panel (*a*) shows the mean performance of NIAViD, along with 95% CIs, as measured by the AUC, *F* measure, precision and sensitivity/recall. The AUC represents the correlation between true-positive and false-positive rates, with values ranging from 0.5 (50%) to 1.0 (100%), where 0.5 (50%) indicates no better than random prediction and 1.0 (100%) indicates perfect agreement between predicted and observed antigenic transitions. The *F* measure is the harmonic mean of the precision and recall. Panel (*b*) shows the ROC curves for the null, training and validation phase performances, with the separate phases marked by distinct colours. The diagonal (broken) line on the ROC reflects theoretical performance that is no better than chance, with uncorrelated antigenic transition status. ROC, receiver operating characteristic.

Our pipeline successfully anticipated the majority of antigenic transitions over this time span. Estimated sensitivity (true-positive rate) on the training data was 88.9% (95% CI, 56.5−98.0%), which compares favourably to the performance on a null model (33.3%, 95% CI, 12.1−64.6%; table 1 and figure 3*a*). (Detailed methods are provided in electronic supplementary material, appendix S1 and text S5.) For comparison, model-detected non-transitions (i.e. true negatives) are comparable to those identified by chance (table 1). Sensitivity on the validation data was 72.7% (95% CI, 43.4–90.3%; table 1 and figure 3*a*). This sensitivity was achieved at a cost in precision (also known as positive-predictive value and is equal to one minus the false-discovery rate), which was estimated to be 12.3% (95% CI, 6.4−22.5%) in the training data and 50.0% (95% CI, 28.0−72.0%) in the validation data (figure 3*a*). An emphasis on sensitivity and prediction can present an incomplete picture of model performance due to the imbalance between the number of antigenic transitions and non-transitions [33]. Hence, we also calculated the area under the receiver operating characteristic curve (AUC), which represents the true-positive rate as a function of the false-positive rate [33] (figure 3*b*). Training and validation AUC for NIAViD are 85.9% (95% CI, 80.4−90.0%) and 79.1% (95% CI, 66.7−87.8%), respectively (figure 3*a,b*), comparing favourably with the null model 51.4% (95% CI, 44.6−58.2%; figure 3*a,b* ).

**Table 1.** The number of cluster transitions to be predicted and four terms used in quantifying the predictions.

	novel influenza A virus detector (NIAViD)		
	null model	training phase	validation phase
transition clusters to predict	9	9	11
TP	3	8	8
FN	6	1	3
FP	62	57	8
TN	132	137	36

The four terms include: TP—the antigenic transitions identified correctly. FN—the antigenic transitions identified incorrectly. FP—the antigenic non-transitions identified as transitions. TN—the antigenic non-transitions identified correctly. The null model for isolation forest and one-class SVM is the model for which the antigenic transitions have been assigned randomly.

FN, false negatives; FP, false positives; SVM, support vector machine; TN, true negatives; TP, true positives.

**Figure 4.**
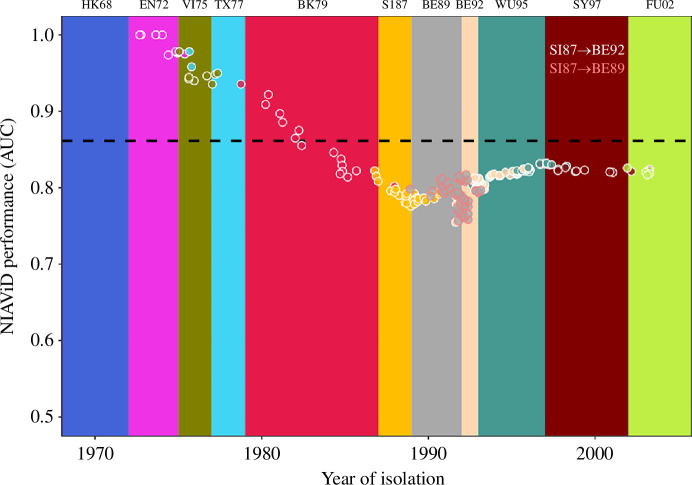
The performance of NIAViD (1968–2002), as measured by the AUC, is plotted against the year of isolation for each virus, stratified by antigenic cluster. The plotted points show the performance as the influenza A virus transitions from one antigenic cluster to another. The white circles represent the transition from SI87 to BE92 (SI87→BE92), while the pink circles represent the transition from SI87 to BE89 (SI87→BE89). In these data, the BE89 cluster does not show any antigenic transition to other strains of the influenza virus. The horizontal (broken) line represents the average AUC of the means for each of the 11 antigenic clusters.

In addition to overall model performance, we examined predictive skill through time. The model exhibited good classification skill in the first decade of the data (AUC > 0.9) followed by a general decrease over time (figure 4), coinciding with two antigenic cluster transitions. The first drop occurred with the extinction of the TX77 cluster [31] and the emergence of the BK79 cluster. The second, a few years later, when two new antigenic clusters, BE89 and BE92, emerged from the SI87 cluster in close succession. Despite these drops, the overall performance of NIAViD was high, with all AUCs exceeding 0.75. This is especially noteworthy given the antigenic bifurcation when SI87 gave rise to BE89 and BE92; the challenge of this event for reliable cluster characterization has been previously noted [6].

To further test the validity of NIAViD, we evaluated its performance using more contemporary data [34]. These data show that recent antigenic clusters have less distinct boundaries (electronic supplementary material, appendix S1 and figure S2), challenging NIAViD even further as it seeks to identify antigenic transitions over time (electronic supplementary material, appendix S1 and figure S3). Unsurprisingly, we see a reduction in both training sensitivity and performance in the validation phase. For the Smith *et al*. [31] data, (1968–2002), training and validation sensitivity are 88.9% (95% CI, 56.5−98.0%) and 72.7% (95% CI, 43.4–90.3%), respectively. For the more recent data from Han *et al*. [34] (1968–2016), training and validation sensitivity are 64.3% (95% CI, 63.6−65.0%) and 56.3% (95% CI, 54.8−57.7%), respectively (electronic supplementary material, appendix S1 and figure S4).

**Figure 5.**
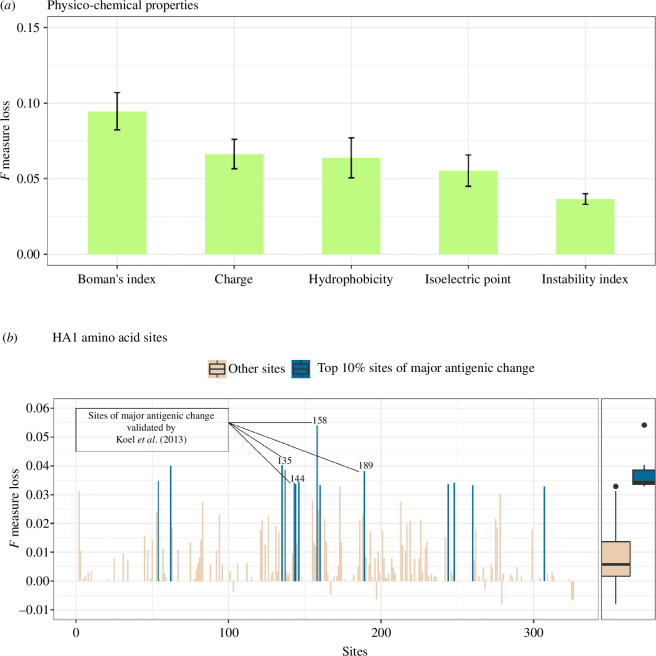
The inferential performance of NIAViD is evaluated using permutation scores. Panel (*a*) shows the *F* measure loss after permuting each of the five physico-chemical covariates, with error bars indicating 95% CIs for the mean *F* measure loss. Panel (*b*) displays the *F* measure loss after permuting 147 out of the 328 amino acid sites associated with the HA1 subregion. These results provide insight into the importance of the physico-chemical covariates and amino acid sites in the performance of NIAViD.

To identify the most important covariates, we quantified the relative importance of each feature to the summary *F* measure using model-agnostic permutation [35] (figure 5*a*). All the physico-chemical covariates have similar importance with mostly overlapping confidence intervals (figure 5*a*), although for both the Smith *et al*. (1968–2002) and Han *et al*. (1968–2016) (electronic supplementary material, appendix S1 and figure S6) data, the Boman’s index is the most important physico-chemical covariate involved in the identification of an antigenic cluster transition in H3N2 viruses (*F* measure 0.09, range 0.08−0.11, figure 5*a*). Additional analysis showed that different physico-chemical covariates could be driving each specific antigenic transition (electronic supplementary material, appendix S1 and figure S7). To identify which specific amino acid sites are most important to cluster transition, we trained NIAViD fitted using one-class SVM and used permutation importance scores to measure how much of the variance in antigenic cluster transition was contributed by each amino acid substitution. Notably, four amino acid sites (i.e. residues 135, 144, 158 and 189) identified as antigenically important by Koel *et al*. [6], and within our top 10% sites of major antigenic change, were found to be statistically more important than other sites (figure 5*b*, boxplot). Most of these major amino sites driving antigenic transitions were also identified in the contemporary dataset (i.e. 1968–20216) (electronic supplementary material, appendix S1 and table S3).

## 3. Discussion

A key challenge for seasonal influenza surveillance and response is the sensitive and rapid identification of novel antigenic clusters. We have shown that antigenic cluster transitions in influenza A (H3N2) viruses can be anticipated from calculable, sequence-based physico-chemical properties and amino acid substitutions in the HA1 protein. In fact, physico-chemical properties outperform (in the training phase), and at least match (in the validation phase) the performance of a model built on the counts of amino acid changes in HA1 (electronic supplementary material, appendix S1 and figure S8). Furthermore, unlike these amino acid counts that depend on a suitable and meaningful reference virus, the physico-chemical properties can be extracted from independent viral isolates. Another notable characteristic of influenza viruses is the glycosylation in the globular head of the HA1 proteins. While these glycosylation sites are important in the immune escape of HIN1 viruses [36], our study indicated no notable relationship between acquisition or loss of glycosylation and antigenic cluster transition in the H3N2 viruses we investigated in this study (electronic supplementary material, appendix S1 and figure S9). A reliable unsupervised tool like NIAViD that detects antigenically novel viral strains could be used to aid vaccine formulation or pandemic preparedness planning. Moreover, NIAViD could serve as a cost-effective complement to deep mutational scanning [37] and reverse genetics [6], streamlining the process by pinpointing specific viral strains for further laboratory testing and validation. NIAViD detected 8 out of the 11 antigenic cluster transitions identified by 2002. The high sensitivity of NIAViD could be valuable for decision contexts where the cost of false negatives is high (e.g. pandemic response). Impressively, the sensitivity estimates obtained with our unsupervised approach are comparable to those reported in studies where supervised learning was employed [4,7–9,38,39]. NIAViD also quantifies the contribution of different biological drivers of antigenic transitions and once we account for specific antigenic transitions, none of the physico-chemical properties individually dominate the transition process. Thus, the global (i.e. full dataset) biological inference might be masking subtle changes in the physico-chemical properties driving individual antigenic transition. Mutations, driving these physico-chemical changes, enhancing the fusion of the full viral HA sequences to cell membranes have been previously identified as novel mechanisms through which the influenza A virus escapes antibody neutralization [25,40]. Also, the importance of the Boman’s index, which measures the protein–protein interaction within the HA1 protein and not with a receptor, suggests that characterization of antigenicity should focus on these interunit interactions especially when interactions with HA2 are considered [41]. Future iterations of this work could further explore these HA2 interactions. Our discovery that certain amino acid sites on the HA1 sequence are highly predictive of antigenic transition is corroborated by Koel *et al*. [8]. However, we also identify additional sites that warrant further investigation (i.e. residues 54, 62, 137, 143, 146, 160, 244, 248, 260 and 307). While these findings provide insight into population averages, we caution that NIAViD does not account for individual-level immune dynamics which are known to play a role in viral antigenicity [42]. One recent study shows how dynamic interactions of immune components may drive cyclic patterns of immunity, including antibodies [43]. Furthermore, within-cluster antigenic differences have been well characterized in previous studies [31] suggesting that other factors, including host immune history and seasonal patterns not captured in our process might also be contributing to viral antigenic evolution. Nonetheless, NIAViD shows that sequence-based approaches to understanding influenza A antigenic evolution can be successful even without antigenic labelling of individual viral isolates.

Supervised learning approaches, such as deep neural network models, have been demonstrated to be effective in accurately predicting HI titres of influenza A (H3N2) viruses using only sequence information [44,45]. We chose to exclude modelled HI titres as NIAViD covariates due to an ongoing shift away from measuring HI titres for virus characterization. Our unsupervised approach with NIAViD, which uses only HA1 sequences, has shown some decrease in performance over time, which may be attributed to changes in the viral population or emergence of new strains not included in the data [23]. In fact, further analysis (electronic supplementary material, appendix S1 and figure S10) shows that NIAViD is robust to historical depth for prediction confirming that the process depends on the signals from all viruses irrespective of the time they were first identified. This suggests the physico-chemical features capture intrinsic antigenic properties rather than time-dependent epidemiological patterns, enabling generalization across historical periods. Thus, re-emphasizing the need to periodically update NIAViD to expand its training data with new strains when they become available. Nonetheless, the model still detects antigenic transitions, even before they persist in the population, further demonstrating that NIAViD is a potentially powerful tool for the detection and characterization of influenza A antigenicity even in the presence of considerable temporal heterogeneity.

As further analysis with the more recent data show, earlier H3N2 antigenic evolution presented punctuated cluster transitions that enabled optimistic model validation, recent years show less-defined groupings with the corresponding decrease in validation performance. With blurring boundaries, binary transition classification becomes more challenging. Assessing incremental viral novelty along an antigenic continuum may be more appropriate in future framework expansions. Nonetheless, NIAViD demonstrated reliable identification of outliers in earlier periods with improved discriminability. Ongoing updates by integrating additional sequenced isolates will likely enhance the detection of emerging viral variants.

Finally, while we fitted NIAViD using both isolation forests and a one-class SVM, other unsupervised learning methods could further improve the performance of NIAViD. For instance, more recent anomaly detection models such as Empirical-Cumulative-distribution-based Outlier Detection warrant future testing in NIAViD [46]. NIAViD can be effectively applied to a wide range of influenza A (H3N2) viral sequences without the need for significant modifications or fine-tuning. Therefore, in future work, we will seek to confirm the robustness of NIAViD using other datasets. Perhaps, models with data-agnostic hyperparameters could improve the performance of NIAViD [47]. Overall, the ability to incorporate different unsupervised learning models is a powerful feature of NIAViD (the pseudocode is provided in electronic supplementary material, appendix S1 and text S3, S4 and S5), as it allows the tool to incorporate improved unsupervised learners as they become available.

In conclusion, as proof-of-concept we have shown that cluster transitions in influenza A (H3N2) antigenicity can be rapidly and sensitively detected using NIAViD without explicitly modelling haemagglutinin inhibition. We suggest that NIAViD be used to help interpret and inform influenza surveillance in both seasonal and pandemic settings.

## 4. Material and methods

### (a) Physico-chemical covariates

Physico-chemical properties of protein sequences can often be associated with these proteins’ functions [24,28,48]. We calculated physico-chemical covariates from HA1 sequences via the seqinr and Peptide packages in R v. 4.2.0 [49,50]. These R packages enable the extraction of physico-chemical properties from individual amino acid sequences. All values were normalized (i.e. 0 mean and unit variance) to ensure that the predicted relationship to antigenic transition was not influenced by the scale of each covariate. We assigned antigenic transition outcomes to each influenza isolate based on these covariates. (See electronic supplementary material, appendix S1 for more information.)

### (b) Data preprocessing and modelling

Influenza A (H3N2) virus HA1 sequence data and cluster identity were obtained from Smith *et al*. [31]. Clusters are named after the first vaccine strain in the cluster and include information about the location and year of isolation, in chronological order HK68, EN72, VI75, TX77, BK79, SI87, BE89, BE92, WU95, SY97 and FU02. Before training models, we stratified the data by antigenic cluster and randomly assigned isolates to the training and validation datasets (see electronic supplementary material, appendix S1 and text S1). We then randomly sampled 80% of isolates without replacement within each antigenic cluster (HK68, EN72, VI75, TX77, BK79, SI87, BE89, BE92, WU95, SY97 and FU02) to create the training set; the remaining 20% from each cluster were retained for validation. Then, isolates belonging to each cluster were ordered by year (the lowest chronological resolution available for the Smith *et al*. data). One isolate randomly selected in the earliest year of each cluster was notionally designated an antigenic transition with other isolates designated as non-transitions (see figure 1 and electronic supplementary material, appendix S1, test S2).

### (c) Anomaly detection

To detect outlying isolates and identify candidates for antigenic transition (i.e. the appearance of a new antigenic cluster), we trained NIAViD by sequentially adding each isolate to the analysis and used isolation forest [17] and one-class SVMs (electronic supplementary material, appendix S1 and text S3) for outlier detection and biological inference, respectively [17] (electronic supplementary material, appendix S1 and text S3). Isolation forest is a method for detecting anomalies that does not use a distance or density measure, while one-class SVM uses a distance metric to learn a hyperplane between normal and anomalous data points [17,51]. The isolation forest performed better at detecting antigenic transitions, so for outlier detection, we present results for NIAViD fitted with isolation forest. In isolation forests, the anomaly score is calculated as the average path length for a sample over all the trees in the forest, with shorter path lengths corresponding to higher anomaly scores [17]. However, to evaluate inferential performance for amino acid substitutions, we fitted NIAViD with one-class SVMs because isolation forest cannot properly handle the one-hot encoded categorical amino acid positional covariates [17]. In one-class SVMs, the raw anomaly score is the distance between a test point and the separating hyperplane, with larger distances indicating more anomalous points. The raw scores are transformed to normalized anomaly scores, using a sigmoid function [51]. The full computational pipeline is illustrated in figure 1.

### (d) Performance evaluation—1968–2002

We evaluated the ability of the framework to identify antigenic cluster transitions by comparing predicted outcomes with known antigenic transitions. To assess the predictive performance relative to a baseline model (electronic supplementary material, appendix S1 and text S5), we generated a null measure of predictive performance by comparing the prediction for each isolate with a randomly assigned antigenic transition outcome. NIAViD allows comparison with a baseline model and quantified the potential of other covariates in informing the antigenic transition of the influenza A virus.

### (e) Performance evaluation—1968–2016

To model antigenic clustering over a more recent period and reflect the evolving mode of HA binding to sialic acid receptors, we applied NIAViD to a published dataset [34] that characterizes influenza A (H3N2) antigenicity beyond 2002. This dataset includes six more antigenic clusters (CA02, BR07, PE09, TX12, SW13 and HK14) that emerged after the Fujian 2002 (FU02) strain. These data contain antigenic coordinates and cluster classifications (electronic supplementary material, appendix S1 and figure S2) for 21 434 isolates. We extracted the physico-chemical properties of these isolates and followed the same data preprocessing and modelling procedures as already described in §4.2. We examined the performance of NIAViD on these data.

### (f) Biological inference

To better understand the biological drivers of our results, we evaluated the inferential performance (i.e. the variance in antigenic cluster transition) of the models using a model-agnostic feature importance score (*F* measure). This score measures the decrease in a model’s score caused by randomly shuffled feature values [35]. We used this feature importance to quantify the contribution of each physico-chemical covariate in explaining the variance in antigenic cluster transition by calculating the *F* measure of each covariate. We also calculated the feature important scores for the amino acid substitutions that inform the physico-chemical changes in the HA1 sequences to further characterize the contribution of these sequences in explaining antigenic cluster transition variance. The predictive importance scores of the top 10% of amino acid sites, identified as major antigenic change sites, were plotted against all other sites using a boxplot in the analysis. Within these top 10% of sites, we highlighted four sites that have been previously validated in the literature [6].

## Data Availability

All data are publicly available from Smith *et al*. [31], Han *et al*. [34] and electronic supporting material, S1 appendix. The data files are publicly available at [52]. The related software files are being hosted by Zenodo at [53]. Supplementary material is available online [54].
